# A Novel 3D-Printed Pediatric Cardiac Model for Teaching Catheterization Anatomy and Access Pathways

**DOI:** 10.7759/cureus.91661

**Published:** 2025-09-05

**Authors:** Joshua A Vasquez, Nathan Vasquez, Jennifer Huang

**Affiliations:** 1 Department of Pediatrics, Oregon Health & Science University, Portland, USA; 2 Pharmacology, Pharmacy Practice – Private Sector, Albuquerque, USA; 3 Department of Pediatric Cardiology, Oregon Health & Science University, Portland, USA

**Keywords:** 3d-printed heart, cardiology education tools, pediatric cardiac catheterization, procedural education model, simulation-based training

## Abstract

Pediatric cardiac catheterization is an essential procedure with limited early trainee exposure and risks of real-time procedural instruction even for late trainees. Traditional teaching tools, such as lectures and diagrams, offer minimal spatial orientation, while commercial simulators are often too costly for widespread use. To address this gap, we developed an anatomically accurate 3D-printed heart model using consumer-grade tools and materials. Anatomically, the model contains hollow lumens running from the femoral arteries through each cardiac chamber and a ventricular septal defect, with a removable front wall for internal viewing. During a structured teaching session, 14 pediatric residents used the model to simulate catheter navigation and identify anatomic landmarks under direct visualization. Post-session feedback (n = 11) showed a mean confidence score increase from 3.45 to 4.27 on a five-point Likert scale. Qualitatively, learners cited improved spatial understanding and procedural clarity. This open-source model provides a cost-effective, reproducible alternative to commercial simulators and may help bridge critical gaps in conceptual training for pediatric cardiac catheterization in resource-limited or early educational settings.

## Introduction

Cardiac catheterization represents a central diagnostic and interventional modality within pediatric cardiology and is foundational to the therapy of a wide range of congenital heart diseases [1]. By providing direct assessment of intracardiac pressures, oxygen saturation, and vascular morphology, it informs decision-making with effects on surgical planning, clinical staging, and late patient outcomes [2-4]. In newborns, urgent catheter-based intervention such as balloon atrial septostomy and ductal stenting can be life-saving [5]. In children, transcatheter therapy can reduce rates of open-heart surgery or serve as an intermediate step in the management of complex lesions such as single-ventricle physiology [3,4]. In addition to procedural roles, catheter-based access assumes a central role within critical care domains, foundational to hemodynamic monitoring, diagnostic assessment, and extracorporeal membrane oxygenation cannulation [6]. While cardiac catheterization plays a central role in pediatric cardiology, residents and early trainees often participate only in perioperative care, with limited exposure to the procedural mechanics and anatomical reasoning of intravascular access [7,8]. This educational gap may persist due to the complexity of procedures, constrained availability of dedicated catheterization labs, and the inherent risks of real-time procedural instruction [8,9]. Observation alone offers limited spatial understanding. Traditional teaching modalities, such as lectures, 2D diagrams, or brief rotations, often fail to convey the procedural pathways and anatomical relationships essential to understanding catheter-based interventions [10]. Simulation-based education presents a promising avenue to address these limitations. It allows trainees to explore procedural steps in a low-risk, controlled environment and has been shown to improve conceptual understanding and confidence, particularly for complex or infrequent procedures such as cardiac catheterization [10-12]; however, many commercial simulators are cost-prohibitive, lack anatomical fidelity, or rely on proprietary systems that restrict use in lower-resource settings [13-15]. To overcome these barriers and promote greater equity in training access, we developed an anatomically informed, transparent heart model using consumer-grade 3D printing tools and affordable materials. The model enables functional simulation of catheter navigation from peripheral vascular access points (e.g., femoral arteries) to central intracardiac targets. It was implemented during a structured teaching session for pediatric residents as part of an introductory curriculum on cardiac catheterization. This technical report outlines the design, construction, and educational integration of this simulation tool, which addresses an important gap in early procedural training and offers an accessible solution for conceptual learning in pediatric cardiac catheterization.

## Technical report

Model design and construction

Digital Design

The anatomic heart model was digitally sculpted using ZBrush (Pixologic, CA), supporting catheter-based navigation along a full vascular trajectory from femoral arteries to central intracardiac structures.

Anatomic Features

**Figure 1:**
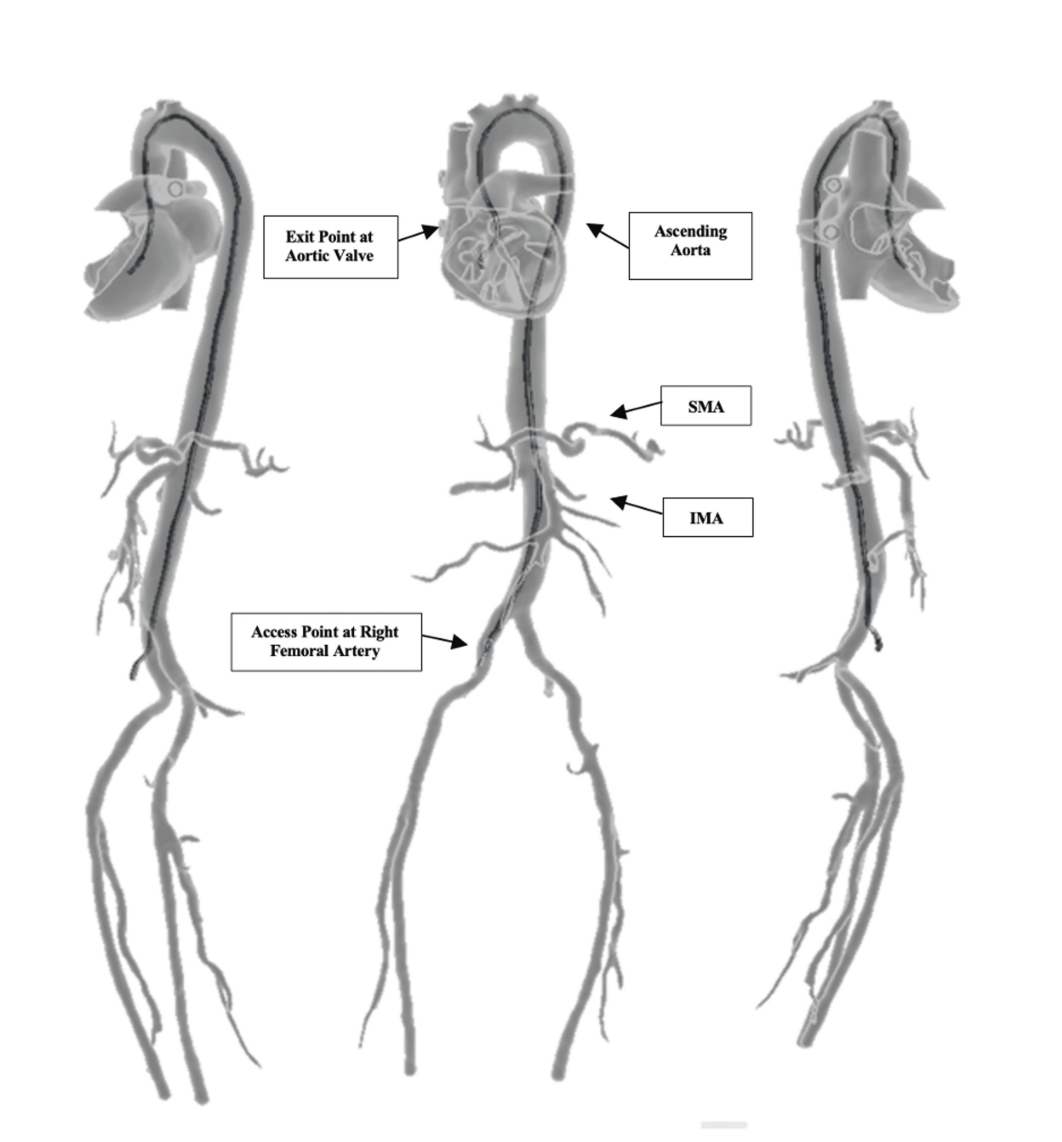
Anterior and lateral views of the 3D-printed vascular model demonstrating peripheral-to-central arterial pathways. Shown are multiple angles of the full vascular trajectory, including bilateral femoral arteries, iliac bifurcation, abdominal aorta, SMA, IMA, thoracic aorta, and aortic arch. These structures allow for simulated guidewire navigation from peripheral to central access points. SMA = superior mesenteric artery; IMA = inferior mesenteric artery

**Figure 2:**
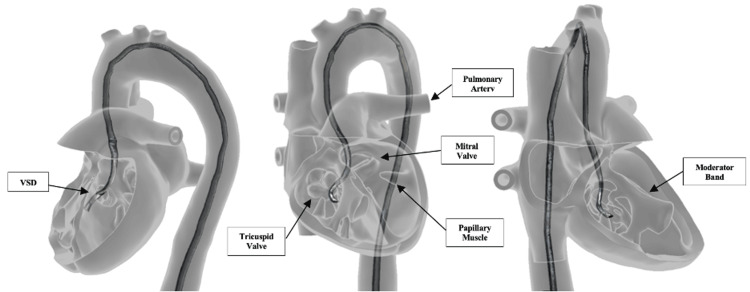
Transparent heart model with intracardiac detail and guidewire trajectory. This figure shows the 3D transparent heart model highlighting internal structures such as the mitral, tricuspid, and pulmonic valves, papillary muscles, moderator band, and a VSD. The guidewire is shown traversing from the aortic arch into both the left and right ventricles, demonstrating simulated intracardiac access. VSD = ventricular septal defect

Anatomically, the model incorporates a complete heart, aortic arch, abdominal aorta, superior and inferior mesenteric arteries, iliac bifurcation, and bilateral femoral arteries (see Figure 1). Intracardiac detail includes static representations of mitral, tricuspid, and pulmonic valves along with papillary muscles and moderator band (see Interactive Model 1 and Figure 2). An aortic valve was intentionally omitted due to creating unrealistic static obstruction and obstructing guidewire passage. A ventricular septal defect was included as a way of allowing learners to practice crossing the interventricular septum with a guidewire. Additionally, the heart’s anterior face was made to be removable for unobstructed visualization of the interior anatomic structures during instruction. All vascular and intracardiac pathways were printed as continuous hollow lumens, permitting complete guidewire advancement from peripheral entry points to intracardiac destinations (see Figures 1 and 2).

**Video 1:** Pediatric cardiac catheterization model. This interactive model displays the heart component. The remaining vascular structures required to assemble the full catheterization model are available through the linked Sketchfab folder.

Slicing and Printing

We prepared the model for printing using Voxeldance Tango slicing software. Printing was completed on the Elegoo Saturn 3 Ultra 12K (Elegoo Inc., Shenzhen, China), a high-resolution masked stereolithography (MSLA) resin printer with an XY resolution of 19 × 24 µ and a build volume of 218.9 × 122.9 × 260 mm. The model was printed with ANYCUBIC Upgraded Standard Clear Resin, a rapid-curing photopolymer resin suitable for high-detail, clear models. The print took around 38 hours total, with a further four hours dedicated to post-processing and final assembly.

Scaling and Assembly

The original model design was 30 inches high, but for educational use, it was scaled by a factor of 1.45, producing a finished assembled model about 44 inches long and 2.58 kg in weight. A visual of the final assembly is provided in Video 1. Printing was done by dividing the model into three main vertical sections (upper, middle, and lower), with each of these sections then further divided into two to five modular pieces for the convenience of 3D printing. Individual pieces were assembled using a UV light-curing methodology (405 nm). Uncured resin was deposited along seam edges and cured into position with a handheld UV light, producing a smooth cohesive form without adhesive or external fasteners.

**Video 1:** Visual of the final model assembly.

Functional setup

Guidewire Construction

To facilitate catheter-based navigation of the printed cardiac model, a cut-to-fit guidewire was constructed with a standard steel guitar string. This material was chosen for its ideal combination of flexibility and stiffness.

Simulation Procedure

Learners inserted the guidewire through a femoral artery entry point on the base of the model. The wire could then be advanced sequentially from this entry point through the iliac bifurcation, abdominal aorta, thoracic aorta, and into the heart. Depending on the procedural goal of instruction, the wire was steered into the left ventricle or guided across a ventricular septal defect into the right ventricle. The translucent walls of the model enabled continuous visual tracking of the guidewire trajectory with real-time spatial orientation during the simulated procedure.

Cost and accessibility

Direct Material Costs

The direct material cost of creating the full-scale catheterization model was approximately $50 USD. This estimate includes 2.5 L of ANYCUBIC Upgraded Standard Clear Resin at approximately $15 per liter, and a handheld UV flashlight with localized resin cure capability at a cost of $13. Other consumables, for example, nitrile gloves and isopropyl alcohol, were used during post-processing and can be considered as general operational supplies consumed on many prints rather than just this model.

Equipment and Software

The model was printed with Elegoo Saturn 3 Ultra, a consumer-grade resin printer currently retailing at approximately $299 USD. Post-processing was completed with a standard resin wash-and-cure station at an estimated $155 USD, although equivalent or makeshift substitutes can be used at a lower cost. Model design was completed within ZBrush for iPad (membership: $9.99/month), and slicing was completed with Voxeldance Tango, included with the printer or separately at $9/month.

Reproducibility

This model design is completely reproducible with commercially available tools and materials. There was no proprietary hardware, advanced level of engineering expertise, or specialized software plug-in use required other than basic 3D design and printing skills. This scalability makes it best suited for educating within medicine outreach projects, resource-limited schooling settings, and early-stage training efforts.

Educational integration

Session Format

A total of 14 pediatric residents participated in the teaching session, representing postgraduate years one through three. To preserve participant anonymity, individual class year data is not recorded. The session was conducted in a small-group format during a scheduled educational block and incorporated both didactic and hands-on components.

Teaching Focus

In the instructional session, learners identified important key landmarks of anatomy along the trajectory as they advanced the wire. Facilitators led participants through the logic of catheter-based access, pointing out anatomical relationships, common routes of navigation, and clinical aspects of procedural planning. The rigidity of the resin construction held its form with only superficial wear observed at the femoral entry point. Advancement of the guidewire was smooth and unobstructed during the session. By combining visual clarity, tactile engagement, and procedural realism, the model offered an effective means of enhancing conceptual understanding and facilitating interactive instruction in catheter-based navigation.

Learner feedback

Survey Results

Of the 14 residents who participated, 11 completed an optional post-session feedback form. The survey assessed self-reported confidence in understanding cardiac catheterization both before and after the session, using a five-point Likert scale ranging from “not at all confident” (1) to “very confident” (5). It also included open-ended prompts regarding the model’s educational value and suggestions for improvement. Before the session, only two (18%) participants rated themselves as “very confident” in their understanding of catheterization. The remaining responses indicated lower confidence levels, with four (36%) learners reporting “somewhat confident,” three (27%) selecting “neutral,” and two (18%) indicating they were “not very confident” or “not at all confident.” After the session, confidence levels increased notably: five (45%) participants reported feeling “very confident,” and four (36%) selected “somewhat confident.” Two (18%) participants remained “neutral,” while no participants selected the lowest confidence categories post-session. The mean confidence score increased from 3.45 to 4.27, reflecting a 24% improvement. Additionally, 9 of 11 respondents (82%) reported an increased confidence level following the session. Due to the limited sample size, inferential statistical analysis (e.g., paired t-test or Wilcoxon signed-rank test) was not performed.

Qualitative Feedback

Qualitative feedback emphasized the model’s usefulness in enhancing spatial understanding and procedural flow. Learners consistently cited the transparent resin, interactive guidewire navigation, and ability to visually track access routes as the most helpful features. The removable anterior wall and the presence of a ventricular septal defect were also highlighted as design elements that enhanced anatomical orientation. Suggestions for improvement included clearer external labeling and the addition of a brief guided walkthrough to complement the hands-on component. One participant noted, “Being able to actually guide the wire helped make sense of the anatomy I’ve only seen on diagrams.” The informal feedback suggested that the model significantly improved learner engagement and conceptual understanding of catheter-based access in a format that was both accessible and memorable.

## Discussion

Educational rationale

This simulation model offers an accessible approach to improving procedural understanding of pediatric cardiac catheterization. Traditional instructional methods, such as lectures, static anatomical diagrams, or echocardiographic video clips, are inherently limited by their 2D presentation and lack of interactivity [16]. In contrast, the 3D-printed, hands-on model presented here provides learners with a spatial and tactile reference for understanding vascular access and intracardiac navigation, thereby enhancing their comprehension of anatomical relationships and procedural logic [11]. Importantly, this simulation model allows for an ex vivo, hands-on approach without the patient risk inherent in real-time catheterization.

Comparison to existing simulators

While several commercial catheterization simulators exist, including systems such as HEARTROID and the Life/form Heart Catheterization Simulator, these platforms are primarily designed for advanced technical skill acquisition rather than introductory conceptual training [17]. Furthermore, their high cost, proprietary hardware, and maintenance requirements often render them inaccessible to smaller academic programs or outreach-based educational efforts [13]. Unlike commercial simulators that arrive as finished products, our model requires digital design, slicing, and assembly steps, but in return offers open-source flexibility and significant cost savings. See Table 1 for a more comprehensive comparison.

**Table 1:** Comparison between the 3D-printed model and a commercial simulator. Data for the commercial simulator column is derived from the Nasco Life/form® Heart Catheterization Simulator product description [18].

Feature	3D-Printed transparent heart model	Nasco Life/form® Heart Catheterization Simulator
Cost	$50 USD (materials only)	$1,680.95 USD
Usability	Designed for conceptual understanding and procedural visualization	Designed for the technical practice of central venous catheterization
Visualization	Transparent resin allows full visualization of internal structures	Synthetic skin with replaceable components; no internal cardiac view
Anatomical targets	Includes heart chambers, great vessels, and a septal defect	Major veins, arteries, and external landmarks only
Interactivity	Allows guidewire navigation and catheter simulation via femoral access	Needle insertion and catheter placement via central venous access (e.g., internal jugular, subclavian) with simulated blood return and anatomical landmark palpation
Portability	Lightweight (2.58 kg), modular, and easy to transport	Heavier (12.09 kg), and includes a hard case
Open source	Object files are freely available for printing	Proprietary, closed design
Educational Focus	Conceptual learning, spatial reasoning, and early procedural exposure	Technical skills (needle insertion, catheter placement)

Purposeful design for accessibility

In comparison, the model described in this report was purposefully designed for anatomical orientation and procedural visualization rather than technical proficiency. Its scalable construction and reliance on consumer-grade tools make it well-suited for integration into early-stage medical education and resource-limited environments. Although 3D-printed heart models are increasingly being used in medical education, most serve as passive visual aids for anatomical demonstration or surgical planning [19]. The provided design files are compatible with consumer resin printers, such as other MSLA or stereolithography systems. With basic adjustments in wall thickness or support structures, they can also be adapted for fused deposition modeling printers, broadening accessibility for institutions with different equipment. To our knowledge, this is among the first open-source, 3D-printed models to offer a fully traversable vascular pathway with real-time visual feedback for catheter-based training.

Limitations

Nonetheless, the model has limitations. All included cardiac valves are static and do not replicate dynamic movement or tactile resistance [19]. No hemodynamic feedback or realistic tension on a catheter exists, and fluoroscopic visualization or technical maneuvers, such as balloon deployment, are not modeled [20]. Its large scale was selected specifically to facilitate educational clarity, yet it does not achieve true anatomical proportions as one would experience during clinical catheterization. Lastly, 3D printing of the model does require basic user knowledge of 3D printing and ultraviolet light curing.

Future directions and distribution

Directions for future development involve exploring the use of this model in more immersive simulation settings, such as C-arm fluoroscopy or augmented reality overlays, as critical next steps to increase procedural fidelity. Additional iterations could incorporate more complex cardiac anatomies to facilitate procedural education for advanced trainees. Longer-term goals include evaluating this model’s educational impact across all levels of medical training, with particular attention to outcomes such as knowledge retention, anatomical comprehension, and procedural planning skills.

## Conclusions

This technical report presents the design, development, and educational integration of a 3D-printed, transparent model created to enhance conceptual understanding of pediatric cardiac catheterization. Pilot testing in a small-group teaching session with pediatric residents demonstrated strong engagement and positive feedback, particularly regarding the model’s clarity and interactivity. Designed with anatomical accuracy, procedural relevance, and ease of assembly in mind, the model supports early-stage learning. The model’s effectiveness in fostering spatial awareness, procedural reasoning, and learner engagement highlights its potential as a valuable supplement to traditional instruction. As simulation-based education becomes more widely adopted, innovations like this can help expand equitable access to foundational procedural training. The files required to print this model will be distributed as a free download alongside this publication, with others encouraged to scale, modify, or adapt the design to suit their institution’s specific educational needs. Constructed entirely with commercially available tools and an open-source workflow, this model can easily be reproduced and presents a scalable solution as a way of bridging early educational gaps within pediatric cardiac catheterization.
